# Long-Term Effects of Hippocampal Low-Frequency Stimulation on Pro-Inflammatory Factors and Astrocytes Activity in Kindled Rats

**DOI:** 10.22074/cellj.2021.7139

**Published:** 2021-03-01

**Authors:** Razieh Rohani, Abbas Aliaghaei, Mohammad-Amin Abdollahifar, Yousef Sadeghi, Leila Zare, Samaneh Dehghan, Mohammad Hassan Heidari

**Affiliations:** 1. Department of Biology and Anatomical Sciences, School of Medicine, Shahid Beheshti University of Medical Sciences and Health Services (SBMU), Tehran, Iran; 2. Department of Physiology, Faculty of Medical Sciences, Tarbiat Modares University, Tehran, Iran

**Keywords:** Deep Brain Stimulation, Epilepsy, GFAP, Interleukin-6, TNF-α

## Abstract

**Objective:**

Epilepsy is accompanied by inflammation, and the anti-inflammatory agents may have anti-seizure effects. In this investigation, the effect of deep brain stimulation, as a potential therapeutic approach in epileptic patients, was investigated on seizure-induced inflammatory factors.

**Materials and Methods:**

In the present experimental study, rats were kindled by chronic administration of pentylenetetrazol (PTZ; 34 mg/Kg). The animals were divided into intact, sham, low-frequency deep brain stimulation (LFS), kindled, and kindled +LFS groups. In kindled+LFS and LFS groups, animals received four trains of intra-hippocampal low-frequency deep brain stimulation (LFS) at 20 minutes, 6, 24, and 30 hours after the last PTZ injection. Each train of LFS contained 200 pulses at 1 Hz, 200 µA, and 0.1 ms pulse width. One week after the last PTZ injection, the Y-maze test was run, and then the rats’ brains were removed, and hippocampal samples were extracted for molecular assessments. The gene expression of two pro-inflammatory factors [interleukin-6 (IL-6) and tumor necrosis factor-alpha (TNF-α)], and glial fibrillary acidic protein (GFAP) immunoreactivity (as a biological marker of astrocytes reactivation) were evaluated.

**Results:**

Obtained results showed a significant increase in the expression of of interleukin-6 (IL-6), tumor necrosis factor (TNF)-α, and GFAP at one-week post kindling seizures. The application of LFS had a long-lasting effect and restored all of the measured changes toward normal values. These effects were gone along with the LFS improving the effect on working memory in kindled animals.

**Conclusion:**

The anti-inflammatory action of LFS may have a role in its long-lasting improving effects on seizure-induced cognitive disorders.

## Introduction

Epilepsy is among the most prevalent brain diseases, has widespread distribution, and about 1% of people suffer from it. Medicinal therapy is the main therapeutic manner in epileptic patients. However, approximately 20- 30% of patients suffer from epilepsy that is resistant to medicinal therapy (1). In addition, about 50% of epileptic patients have variable degrees of cognitive impairments that seriously influence the quality of life of patients (2). Therefore, there are a lot of efforts to find new therapeutic methods to reduce the severity of seizures in these patients. 

Application of deep brain stimulation (DBS) is a possible treatment for drug-resistant epileptic patients. U.S. food and drug administration (FDA) has approved the DBS applying in some brain areas, including anterior thalamus and hippocampus, as a new therapy in epileptic patients (3). The pattern of DBS, especially its frequency, is an important factor in its effectiveness. DBS has been applied in a wide range of frequency (from 1-190 Hz) in epileptic patients [reviewed in: (4)] and laboratory animals (5-10). Applying DBS at low-frequency (named low-frequency stimulation; LFS) exerts anticonvulsant effects. Interestingly, the neuronal damage induced by low-frequency stimulation (LFS) application in the epileptic and its surrounding areas is less than the damage caused by high-frequency stimulation. Accordingly, LFS may be considered as an appropriate choice for epileptic patients (11). In addition to its anticonvulsant effect, LFS restores the learning and memory impairment following seizures (12, 13).

Finding the precise mechanisms of antiepileptic and anticonvulsant actions of DBS is required before its application as an anticonvulsant method. Different mechanisms, such as changes in neuronal excitability and gene expression, have been suggested to be involved in DBS anticonvulsant effects (14). In addition, recently, it has been reported that high-frequency stimulation of thalamus has an anti-inflammatory effect in epileptic models (15). The plasma concentrations of IL-1, TNF-α, and IL-6, as important inflammatory agents, are increased in individuals with temporal lobe epilepsy. These agents have also detected from resected brain tissue from people with intractable epilepsy (16). In line with the role of inflammatory agents in epilepsy, the induction of IL1α, TNF-α, and IL-6 has been shown in both neurons and glia before epilepsy onset. There is an increase in inflammatory cytokines levels (IL-1β, IL-6, and TNF-α) in the hippocampus of PTZ-induced kindling (17). Inflammatory agents increase in a laboratory model of seizure, including PTZ kindling. It has been shown that PTZ-induced kindling causes significant neuronal injury and expression of the pro-inflammatory TNF-α in the cerebral cortex (18).

It is hypothesized that focal or systemic impaired inflammatory processes lead to unusual connectivity in the central nervous system and may increase the excitability of neuronal networks. Therefore, inflammation can mediate the onset of epilepsy (19). Instead, it has been reported that the LFS application reduced the seizureinduced hyperexcitability (20). Considering the fact that, PTZ kindling profoundly affects the hippocampus, and the hippocampus is a vital structure for the acquisition of new memories (21), in the present study we applied LFS in the CA1 region of the hippocampus (as an important area in generation and propagation of kindled seizures). Then we investigated the effect of LFS on inflammatory factors and working memory following PTZ kindling in rats.

## Materials and Methods

### Animals

Male Sprague-Dawley rats (weighed 200-220 g) were used in the present experimental study. Animals were prepared from the animal house of Shahid Beheshti University of Medical Sciences. All experiments and research protocols were in accordance with the guidelines established by the Animal Care Commission of Shahid Beheshti University of Medical Sciences (the ethical approval number was: IR.SBMU.MSP.REC.1395.447). All efforts performed to reduce the pain and discomfort during experiments. Animals were caged in groups of four and had free access to food and water. The lightdark cycle was adjusted for 12-hours (lights from 07:00 A.M. to 7:00 P.M.), and the temperature was controlled in the range of 22-24˚C. The number of animals and their distress were kept minimized during the experiments.

### Experimental procedure

The animals were divided into intact, sham, LFS, kindled (K), and kindled +LFS (KLFS) groups. In the kindled group, animals received PTZ until three sequential stages 4 or 5 seizures were observed, and these animals were considered as fully kindled. A similar protocol was performed for the animals in the KLFS group; however, after achieving the fully kindled state, animals received LFS at four-time points. LFS was applied in LFS group animals similar to the KLFS group but did not receive kindling stimulation. In the sham group, the animals underwent a surgical procedure, without receiving LFS or kindling stimulations. The intact group did not undergo any surgery, LFS, or kindling stimulations.

### Animal surgery

Rats were anesthetized by intraperitoneal injection of ketamine and xylazine (100 and 10 mg/kg, respectively) before surgery. The animal head was fixed in a stereotaxic instrument. A tripolar stimulating/recording electrode was implanted into the hippocampal CA1 region of the right hemisphere coordinated as follows: 3.2 mm posterior and 2 mm to the right from bregma and 2.3 mm below dura (22). The electrode consisted of twisted Tefloncoated stainless steel strands, insulated except at their tips, with a diameter of 127 μm (A-M Systems, USA). Three miniature stainless steel screws were also fixed on the skull to secure the electrode assembly. One screw was connected to an insulated stainless steel wire and served as a monopolar ground and reference electrode. Implanted electrodes were attached to pins of a small plastic multichannel socket. The plastic socket was attached to the skull with dental acrylic as a head stage.

### PTZ kindling procedure

Chemical kindling was induced by intraperitoneal injection of a sub-threshold dose of PTZ (34 mg/Kg; 0.1 ml/100 g) every other day. The convulsive behaviors of each animal were observed immediately following PTZ injection for 20 minutes when the rat was put in a transparent plexiglass box (30×30×30 cm). The Seizure intensity was evaluated using a modified Racine scale. In stage 0, no response was observed. In stage 1, ear and facial twitching occurred. Stage 2 was distinguished by convulsive twitching axially through the body. In stage 3, rats showed myoclonic jerks and rearing. Stage 4 was accompanied by wild running and jumping, and finally, in stage 5, generalized tonic-clonic seizures were observed (23). PTZ was dissolved in sterile isotonic saline as a vehicle exactly before the injections. In the sham group, animals received the vehicle and were handled similar to the animals of the kindled group. All animals weighed before each injection. 

### Low-frequency stimulation application

LFS was administered at four different time points. The 1^st^ LFS was applied at 20 minutes, and the 2^nd^ LFSs was applied at 6 hours after the last PTZ injection. The 3^rd^ and 4^th^ LFSs were applied the next day at the same time (i.e., there was a 6-hour interval between third and fourth LFSs). Each LFS contained four trains of 200 square monophasic pulses at 0.1 ms duration and 1 Hz. LFS trains were applied at 5 minutes intervals. The LFS intensity (200 μA) adjusted according to previous experiments (12). Using a PC-based stimulating and recording system (D3111 ScienceBeam instrument Co., Iran), LFS parameters were determined. During LFS administration, local field potentials were recorded from the hippocampal CA1 using a custom-designed software, eTrace analysis (version 2 ScienceBeam instrument Co., Iran), to confirm the LFS pulses were applied at the site. 

### Y-maze test

The spatial working memory was assessed by the Y-maze test. The apparatus had three arms separated by 120° angles. Each arm was made of black Plexiglass (30 cm long × 8 cm wide × 15 cm high). There were also different cues outside the maze to make different spacial properties for each arm. Each animal was randomly placed in an apparatus arms and could freely explore the maze for 5 minutes. The consecutive entrance of animals (without repetition) into three different arms was considered as a spontaneous alternation. The spontaneous alternation percentage was measured as the ratio of actual (total alternations) to possible (total arm entries-2) number of alternations × 100.

### Quantitative real-time polymerase chain reaction

The expression of IL-6 and TNF-α genes was measured by quantitative real-time polymerase chain reaction (qRTPCR). One week after the last PTZ injection, animals were anesthetized with CO_2_ , sacrificed, and their dorsal hippocampi were isolated and preserved in RNAlater solution at −20˚C. According to the manufacturer’s instructions, we used the High Pure RNA Tissue Kit (Roche, Basel, Switzerland) to extract total RNA. In the presence of random hexamers and RNase inhibitor, 1 µg of total RNA was transcribed to cDNA using murine leukemia virus (MuLV) reverse transcriptase (Fermentas, Lithuania). The qRT-PCR analysis was run using specific primers for IL-6 and TNF-α genes. GAPDH & ribosomal RNA 18s were used as internal controls (Table 1). Reactions were performed using SYBR® Premix Ex Taq™ II (TAKARA BIO INC.) on a Rotor-GeneTM 6000 real-time PCR machine (Corbett Research, Qiagen, Germany). Initial denaturation was performed at 95˚C for 15 minutes. Then, 40 denaturation cycles were run at 95˚C for 5 seconds, under primer specific conditions (Table 1), and extension at 60˚C for 20 seconds. Comparative qRT-PCR quantitation was performed between candidate groups using REST 2009 (Relative Expression Software Tool, Qiagen). 

### Immunofluorescence investigations

Animals were anesthetized with CO_2_ , sacrificed, and their brain was removed for Immunofluorescence study at one week after the last kindling stimulation. The paraffin blocks of the brains were processed and sectioned by a Leica semi-motorized rotary microtome (Leica RM 2145, Germany) with 10 µm thickness. The slides with tissue sections were immersed into xylene (3 changes, 10 minutes each), and were transferred from xylene into 100% ethanol (3 changes, 10 minutes each). Then, they were immersed into 95, 80, and 70% ethanol (1 change, 5 minutes each). At the next step, slides were immersed in a retrieval solution (sodium citrate, pH: 6) jar and autoclaved at 95˚C for 20 minutes. Then, they were let cool to room temperature for 25-30 minutes and were transferred into washing buffer (phosphate buffer solution; PBS). 0.2% Triton X-100 was used to make the samples permeabilized. Then, samples were blocked with 10% normal goat serum for 1 hour. The sections were incubated with chicken anti-GFAP primary antibodies (aVeS Co.; USA) overnight at 4˚C. After extensive washing with PBS and 1-hour incubation with an appropriate fluorescent-labeled rabbit anti-chicken IgY H & L (Cat No: ab6751; Texas Red@) secondary antibody. The prepared samples were washed with PBS. Tissue sections were counterstained with 4,6-diamidino-2-phenylindole (DAPI) as a nuclear dye and were coverslipped, then examined under a fluorescence microscope. To quantify the immunostaining data, we used the ImageJ software. The mean gray value of the desired area was subtracted by the mean gray value of the background.

**Table 1:** Sequences of primers used in real-time polymerase chain reactions


Primer Name	3´-Sequence-5´	Annealing	NCBI Accession number

18s rRNA	F: GAGAAACGGCTACCACATCC	55˚C × 25	NR_046237.1
	R: TTTTTCGTCACTACCTCCCC	second	
GAPDH	F: GAACATCATCCCTGCATCCA	60˚C × 25	NM_017008.4
	R: GCCAGTGAGCTTCCCGTTCA	second	
IL-6	F: TCTCTCCGCAAGAGACTTCCA	55˚C × 25	NM_012589.2
	R: ATACTGGTCTGTTGTGGGTGG	second	
TNF α	F: ACCACGCTCTTCTGTCTACTG	60˚C × 25	NM_012675.3
	R: CTTGGTGGTTTGCTACGAC	second	


IL-6; interleukin-6, TNF-α; Tumor necrosis factor- alpha, and GAPDH; Glyceraldehyde-3-phosphate dehydrogenase.

### Statistics

Statistical analysis was done using GraphPad Prism version 6.01 for Windows (GraphPad Software, Ca, USA). Data were averaged and expressed as mean ± SEM. The normality of distribution of data was checked by the Kolmogorov-Smirnov test, and the p-values were calculated for all experimental groups. Obtained results showed the normal distribution of data. To evaluate the effect of kindling and LFS application on different parameters in experimental groups, one-way ANOVA was used, followed by Tukey’s post hoc test. The values of spontaneous alternation in all groups were also compared with a chance level of 50% by using a one-sample t test. P-value of less than 0.05 was considered to represent a significant difference.

## Results

Animals showed fully kindled seizures (i.e., the consecutive stage 4 or 5 seizures) after receiving 10.44 ± 1.04 PTZ injections in kindled and after receiving 11.33 ± 0.91 PTZ injections in kindled+LFS groups. There was no significant difference in the kindling rate between these two groups, showing similar neuronal excitability in the animals of these two groups. As there was no significant difference in intact and sham groups, their data were merged and were considered as the control group. In addition, previous experiments showed that the applied pattern of LFS exerted an anticonvulsant effect in fully kindled animals (24, 25).

**Fig.1:**
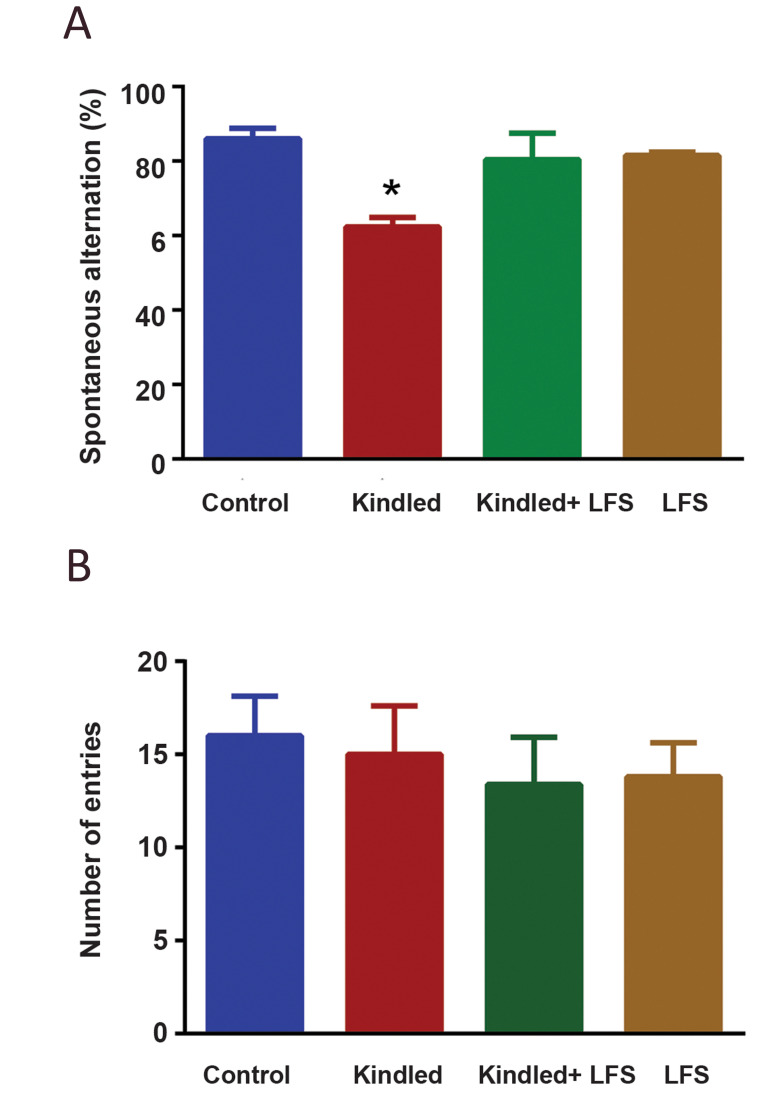
Effect of low-frequency stimulation (LFS) on kindling-induced impairment in working memory.** A. **Spontaneous alternations were measured as an index of working memory in rats. Applying LFS in kindled animals restored the reduction of spontaneous alternations at one week post its application. LFS alone had no significant effect on this parameter. **B.** There was no significant difference in the number of entries between experimental groups. *P<0.05 when compared to control group. Data are presented as mean ± SEM (Control n=6, LFS n=5, Kindled n=7, Kindled+LFS n=6).

When the working memory was evaluated in fully kindled animals (n=7) at one week after the last PTZ injection, there was a significant (P<0.05) reduction in their spontaneous alternation compared to the control group (n=6, Fig .1A). Applying LFS in fully kindled animals restored the working memory impairment. There was no significant difference in spontaneous alternation between kindled + LFS (n=7) and control groups (Fig .1A). Administration of LFS alone (n=5) had no effect on working memory. In addition, there was not any significant difference in the number of total entries among experimental groups in the Y-maze test (Fig .1B). 

**Fig.2:**
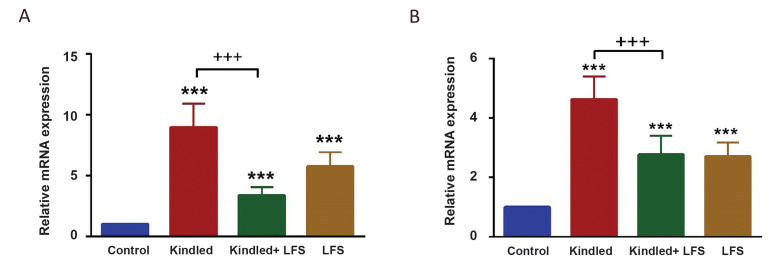
Effect of low-frequency stimulation (LFS) on kindling-induced increment in pro-inflammatory factors. **A.** Tumor necrosis factor- alpha (TNF-α) and **B.** interleukine -6 (IL-6) were significantly increased in kindled animals. Applying LFS in kindled animals reduced the gene expression of TNF-α and IL-6 at one week post its application significantly. LFS alone had also significant effect on these parameters. ***P<0.001 when compared to control group and +++ P<0.001 compared with the related group. Data are presented as mean ± SEM (Control n=3, Kindled n=3, KLFS n=3, LFS n=3). mRNA; Messenger ribonucleic acid.

In the next step, we tried to find the effects of LFS on inflammatory mediators. The gene expression of both TNF-α and IL-6 increased in the hippocampus of kindled animals (n=3; P<0.001). When LFS was applied in full kindled animals (kindled + LFS group; n=3), there was a lower increase in the expression of these genes compared to the kindled group, and there was a significant difference between kindled and kindled + LFS groups (P<0.001). However, there was a significant increase in the gene expression level of both TNF-α and IL-6 in kindled + LFS compared to control animals (P<0.001; Fig.2). Thus, LFS could not completely return the level of gene expression of TNF-α and IL-6 toward control situations. Interestingly, while LFS reduced TNF-α and IL-6 gene expression in kindled animals, applying LFS alone (n= 3) in the control group significantly increased the expression of these two genes (Fig .2).

**Fig.3:**
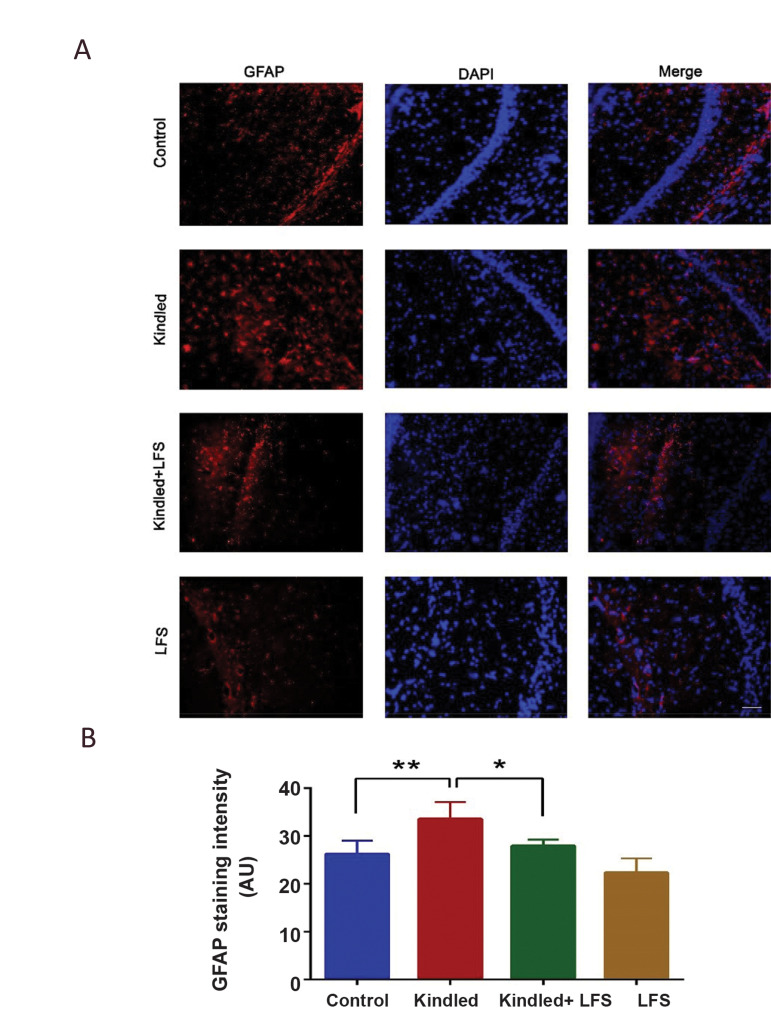
Effect of low-frequency stimulation (LFS) on kindling-induced increment glial fibrillary acidic protein (GFAP). **A. **Representative immunofluorescence images for GFAP (red), nucleus (DAPI, blue), and merged in the hippocampal CA1 subfield.** B.** Quantification of GFAP signals in different experimental groups. GFAP was significantly increased in kindled animals. Applying LFS in kindled animals reduced GFAP expression at one week post its application significantly. LFS alone had no significant effect on these parameters. *P<0.05 and **P<0.01 when compared to control group. Data are presented as mean ± SEM (Control n=5, LFS n=4, Kindled n=6, KLFS n=4).

To confirm the effect of LFS on seizure-induced changes in the inflammatory system, we also compared the amount of glial fibrillary acidic protein (GFAP) in different experimental groups by immunofluorescence method. Obtained results showed a significant increase in the expression of GFAP in the hippocampal CA1 area of the kindled animals (n=6; P<0.01). The application of LFS in the CA1 region decreased the expression of GFAP compared to kindled group (P<0.05). There was no significant difference between kindled+LFS (n=4) and control (n=5) groups (Fig .3).

## Discussion

Obtained results demonstrated that LFS applying in the hippocampal CA1 region of full PTZ kindled rats had a long-lasting effect and reduced the inflammatory agents in the hippocampus at one-week post kindled seizures. It was previously shown that the LFS pattern used in the present study had an anticonvulsant effect on the kindled animals (25, 24). 

There is a strong relationship between epilepsy and inflammation, and recently, the anti-inflammatory agents are thought to reduce and control the seizure attacks (26), although it is not completely clear whether inflammation causes epilepsy or is a result of epilepsy. Similar to previous reports, our data showed a significant increase in inflammatory factors, including IL-6 and TNF-α. During seizure development, the production of IL-6 and TNF-α is increased significantly (27). The increment of IL-6 and TNF-α may be involved in the epileptogenesis via different mechanisms including exerting a modulatory effect on glutamatergic transmission (28), potentiating the function of N-methyl-D-aspartic acid (NMDA) receptors via activation of non-receptor tyrosine kinases (29), and changing the synaptic transmission through GABAergic neurons (30). Therefore, the decrement of inflammatory agents may be partly considered as a mechanism of the anticonvulsant effect of LFS. Of course, it must be considered that the expression levels of IL-6 and TNF-α genes were related to both hippocampal neurons and glial cells.

In line with the results of the present study, it has been reported that the application of deep brain stimulation exerts anticonvulsant and anti-inflammatory effects (31). However, there are many differences between these studies and ours: a. in these studies the researchers used high-frequency stimulation (130 Hz at the intensity of 400 μA) while we used LFS at lower intensity (1 Hz at the intensity of 200 μA); b. they stimulated the anterior nucleus of thalamus while we stimulated the CA1 region of the dorsal hippocampus and c. they measured the changes in inflammatory factors while stimulation was switched on. However, in the present study, we assessed the inflammatory agents at a one-week post-LFS. On the other hand, we evaluated the long-lasting effect of LFS on the brain inflammatory system. Thus, considering the fact that the amount of neuronal damage in response to LFS is less than damage resulted from high-frequency stimulation (11). LFS may be suggested as a better pattern of stimulation in epileptic patients.

Changes in the expression of GFAP also confirmed the protective effect of LFS on the inflammatory system in kindled animals. GFAP is expressed by and is an index of astroglial activation. Epileptic seizures lead to an increment in GFAP expression in different brain areas, including the hippocampus (32). In addition, astrocyte dysfunction contributes to the generation or spread of seizure activity. Accordingly, astrocytes should be regarded as important targets for the new alternative antiepileptic strategies, including deep brain stimulation (33). Our present study showed that LFS applying in fully kindled animals restored the GFAP expression toward its normal values. Of course, as we showed the fluorescent intensity and not the number of cells (neurons and glia) in the immunostaining experiment, the probable changes in neuronal numbers in different experimental groups may be related to an increase in GFAP expression. Therefore, it is better to count the number of cells in future experiments.

The increment in the activity of astrocytes, and therefore over-expression of GFAP can be observed in many brain diseases. In the first step, the activation of astrocytes may protect the brain through different mechanisms such as repairing the blood-brain barrier, limitation of the damaged area, and the release of neurotrophic factors (34). However, following their activation, astrogliosis has neurotoxic effects and increases the progression of the disease, since it exacerbates the inflammatory reactions through producing the cytokines and promoting the glutamate release (35). These mechanisms may be suggested to exacerbate of seizure-induced brain damage. Accordingly, reducing the biological activity of astrocytes following the LFS application may have a role in the long-lasting protective effects of LFS. Of course, it must be emphasized that, considering the growing data about the impact of the glial cells in the mechanisms of the DBS therapies, more studies needed to find the time-course of the brain tissue inflammatory reaction following deep brain stimulation (36).

The activation of astrocyte is regulated and be controlled by many factors, including IL-6 and TNF-α (37). Therefore, the observed increase in IL-6 and TNF-α in our study are in line and can be considered as a reason for increasing of GFAP expression. On the other hand, the inhibitory effect of LFS on GFAP may be due to its inhibitory effects on these pre-inflammatory factors, but not its direct effect on astrocytes themselves. It must be considered that other important cells involved in brain inflammation are microglial cells. Therefore, it is recommended to measure Iba-1 (as a molecular index of neuroglial activities) in future research. 

Considering that the ameliorating effect of LFS is applied through different mechanisms, it cannot be concluded from the presented results that whether LFS directly affects inflammatory responses or it influences them indirectly by modulating the neurotransmitter and/ or neuromodulatory systems.

Another finding of the present study was the restoring effect of LFS on working memory in kindled animals. This finding was in line with our previous study in which the application of LFS had an improving effect on working memory at 24 hours after the last kindling stimulation (12). However, our present study confirmed that this improving effect lasts for at least one week after the last kindled seizures. Many experimental models of seizures are accompanied by cognitive abnormalities. In addition, many epileptic patients have also memory impairment (12, 38).

Many factors can be considered as the mechanisms involved in these kinds of memory impairments; however, one probable reason for these comorbidities may be the chronic activation of inflammatory agents. Some investigators showed that the increase in cytokines and increment of their signaling resulted in memory impairment, and there are many reports about the role of inflammatory cytokines, such as IL-6 and TNF-α in the molecular mechanisms underlying learning and memory consolidation (39). Our data showed that LFS’ improvement of working memory was accompanied by decreasing the inflammation in rat brains. Thus, obtained results are in line with the previous studies suggesting the seizure-induced inflammatory factors may potentially be involved in memory impairment following seizure behaviors. 

In our experiments, the animals were chronically implanted with the electrodes. Therefore, it may be suggested that the inserted electrodes were partly the reason for inflammation in the brain of rats. It has been shown that the implanted electrodes, used for deep brain stimulation, result in glial scars. However, this damage is restricted to a very small area in nearby the electrodes (40). Therefore, all of the observed changes in inflammatory actions in the present study can be related to seizure induction. 

## Conclusion

Obtained results showed that LFS applying in the dorsal hippocampus of kindled animals reduced the seizure-dependent inflammatory reactions and restored the memory impairment at a long-lasting time (one week) post-seizure. This protective effect was observed both in the gene expression of pro-inflammatory factors and astrocyte activation and in working memory as an important cognitive behavior. However, more studies need to shed light on the precise mechanisms of LFS, finding the best pattern of LFS and the best brain region of stimulation. 
